# Effects of physical activity on depression, anxiety, and stress in college students: the chain-based mediating role of psychological resilience and coping styles

**DOI:** 10.3389/fpsyg.2024.1396795

**Published:** 2024-06-07

**Authors:** Ming Liu, Huanju Liu, Zhuzhu Qin, Yining Tao, Wan Ye, Renyang Liu

**Affiliations:** ^1^Department of Public Physical and Art Education, Zhejiang University, Hangzhou, Zhejiang, China; ^2^Center for Genetic Medicine, The Fourth Affiliated Hospital, Zhejiang University School of Medicine, Hangzhou, Zhejiang, China; ^3^School of Nursing, Fujian University of Traditional Chinese Medicine, Fuzhou, Fujian, China; ^4^Department of Global Public Health, Karolinska Institutet, Stockholm, Sweden; ^5^Department of Nursing, Xiamen Medical College, Xiamen, Fujian, China; ^6^Emergency and Critical Care Center, Intensive Care Unit, Zhejiang Provincial People's Hospital (Affiliated People's Hospital), Hangzhou Medical College, Hangzhou, Zhejiang, China

**Keywords:** physical activity, negative emotions, psychological resilience, coping styles, chain mediation

## Abstract

**Background:**

Physical activity can alleviate negative emotions in college students by enhancing mood and cognitive functions. Yet, the mechanisms underlying these benefits remain unclear.

**Purpose:**

This study examines the association between physical activity and negative emotions—specifically, depression, anxiety, and stress—in college students. Additionally, we explore the mediating effects of psychological resilience and coping styles to offer theoretical and practical insights for mitigating students’ negative emotions.

**Methods:**

Using a stratified random sampling approach, a total of 1,380 college students, from five universities in Hangzhou, Nanjing, and Wenzhou, were included in the analysis. The survey instruments included the Physical Activity Rating Scale, Connor-Davidson Resilience Scale, Simple Coping Style Scale, and Depression-Anxiety Stress Scale. The data were statistically analyzed using multivariate methods with IBM SPSS 25.0 and the PROCESS V3.3 plug-in.

**Results:**

(1) College students engage in a low level of physical activity, with male students participating significantly more than female students (*p* < 0.001). (2) Physical activity was significantly positively correlated with psychological resilience and positive coping styles (*t* = 9.126, *p* < 0.001; *t* = 23.087, *p* < 0.001) and overall negative correlated with negative emotions in college students (*t* = −3.601, *p* < 0.001). (3) Psychological resilience and positive coping styles were found to play a chain mediating role between physical activity and negative emotions. The mediation effect consists of two paths: physical activity → psychological resilience → negative emotions (effect value: −0.0324), and physical activity → psychological resilience → positive coping → negative emotions (effect value: −0.0099). (4) Female students demonstrated higher levels of positive coping (*p* < 0.001), while male students exhibited more negative emotions (*p* < 0.001).

**Conclusion:**

Our study identifies a significant indirect link, mediated by psychological resilience and positive coping styles, between physical activity and the reduction of negative emotions. Targeted interventions addressing gender differences, such as offering special courses and providing specialized exercise programs and emotional management strategies, can enhance psychological resilience and positive coping mechanisms. Consequently, these measures can alleviate the adverse effects of negative emotions. Our findings have broader implications for both research and practical interventions in promoting mental health among college students.

## Introduction

Negative emotions, a common and serious problem among college students, can affect their mental and physical health, academic performance, and quality of life (Yousif et al., 2022). The sources of negative emotions in college students are varied and complex, including academic pressure, competition, social stress, emotional issues, and financial strain (Karyotaki et al., 2020). These stressors can trigger negative emotions and impair cognitive functions, such as attention, memory, and decision-making (Gong et al., 2023; Wen et al., 2023). A national Healthy Minds Study found that over 60% of students were dealing with at least one mental health issue, such as symptoms of depression and self-injury (Lipson et al., 2022). This study also revealed significant disparities across the US, with these issues being particularly prevalent among racial/ethnic minority students. Further studies have underscored a positive correlation between pressure and depression among college students, with the prevalence of depression among Chinese college students reported to be as high as 28.4% (Gao et al., 2020). Long-term exposure to negative emotions can not only lead to mental disorders but also physical problems such as inflammation and cardiovascular diseases (Schneiderman et al., 2005). Therefore, the mental health of college students has become a social issue warranting attention from all sectors of society.

One of the factors that can influence college students’ mental health is physical activity. Insufficient physical activity among college students can compromise bone and functional health, potentially leading to obesity, vision loss, and serious psychological issues (Brown et al., 2024). Physical activity can confer numerous benefits to both the body and mind, including mood enhancement, improved self-confidence, alleviation of mild depression and anxiety symptoms, better concentration, enhanced learning engagement, and memory boost (Malm et al., 2019; Li and Guo, 2023). Moreover, physical activity can also reduce the level of cortisol, a stress hormone, and improve the immune system (Gerber et al., 2020; Xu et al., 2021). Physical activity serves as a beneficial coping mechanism for negative emotions, especially during the quarantine period caused by the COVID-19 pandemic (Xu and Yang, 2023). Some studies have demonstrated a negative correlation between moderate physical activity and negative emotions in college students (Aperocho et al., 2021; Tao et al., 2023). In addition, physical activity can also improve the academic performance of college students by reducing their academic anxiety (Fricke et al., 2017).

However, the relationship between physical activity and negative emotions in college students may not be as straightforward or simple as it appears. There may be underlying mechanisms that mediate or moderate this relationship. For example, some studies have suggested that psychological resilience and coping styles are important factors that affect how college students deal with negative emotions (Freire et al., 2020; Wu et al., 2020). Psychological resilience is characterized as the ability to rebound from substantial stressors, including familial and relational challenges, serious health issues, and occupational or financial difficulties. In the face of these stressors, coping styles are the strategies employed to manage and adapt to such adversity. Given this, it is conceivable that physical activity may exert an indirect influence on negative emotions by modulating psychological resilience factors and coping styles. This underscores the need for a deeper investigation into the pathways by which physical activity mitigates negative emotions among college students.

Psychological resilience is considered an important protective factor in mitigating adversity and promoting the healthy development of individuals (Kim and McKenzie, 2014; Moeller et al., 2020). As renowned educator Cai Yuanpei said, “To develop a sound personality, one must start with physical education.” This suggests that physical education helps build qualities like strength, perseverance, bravery, self-confidence, and a strong spirit when facing challenges. These qualities are closely linked to psychological resilience, a positive trait that helps individuals face, overcome, and learn from stressors (Fricke et al., 2017; Aperocho et al., 2021). Previous studies have shown a significant correlation between physical activity and enhanced psychological resilience in college students (Xu et al., 2021; Li and Guo, 2023). Thus, incorporating physical activity into their daily routines could be a promising approach to fostering resilience and promoting mental health.

Coping styles, another factor influencing how individuals manage stress, are the cognitive and behavioral efforts an individual makes to mitigate the negative effects of stress. Cognitive Appraisal Theory (CAT) Model, proposed by Richard S. Lazarus, suggests that humans can resolve dilemmas they face through psychological adjustments and effective coping efforts to reduce stress levels (Biggs et al., 2017). This theory underscores the proactive role individuals can play in managing their stress responses, highlighting the interplay between cognitive processes and emotional regulation. Coping styles are usually classified as positive and negative coping (Moeller et al., 2020). Research has shown that systematic training in physical dance can foster more positive coping styles and promote a healthier psychological state among college students (Bayram and Bilgel, 2008). Thus, through cognitive appraisal and the adoption of positive coping styles, potentially fostered by activities such as physical dance, individuals can effectively manage stress, enhancing their psychological well-being and overall quality of life.

Psychological resilience and positive coping styles are both positive psychological traits. Psychological resilience has been found to predict the adoption of positive coping styles and inversely predict negative ones (Blanco et al., 2008). Individuals exhibiting high psychological resilience tend to adopt positive coping styles and are more proactive in addressing and resolving negative emotions (Gong et al., 2023; Xu and Yang, 2023). Therefore, the mechanism through which physical activity affects negative emotions may be attributed to the influence of psychological resilience and coping styles on college students’ negative emotions.

**Figure 1:**
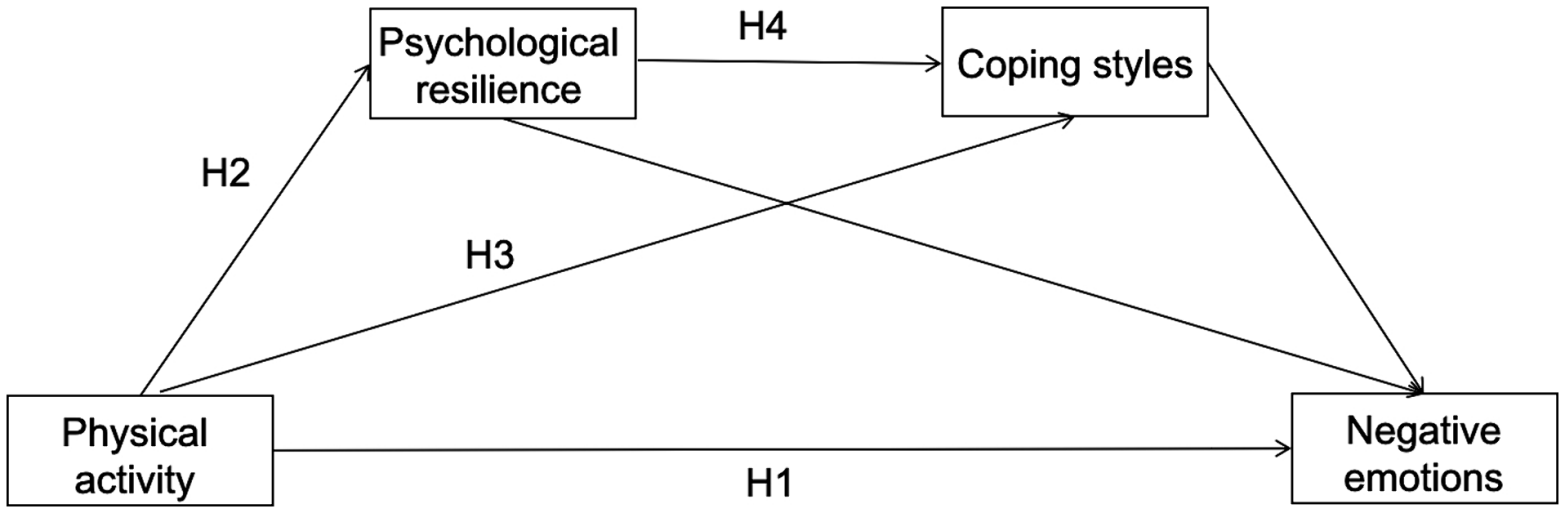
Hypothetical model proposed in this study.

In summary, our study investigates the impact of physical activity on negative emotions, namely depression, anxiety, and stress, among college students. We aim to explore the mediating roles of psychological resilience and coping styles in this relationship. Specifically, we examine how psychological resilience mediates the relationship between physical activity and negative emotions, how coping styles mediate this relationship, and how psychological resilience and coping styles act as chained mediators. Chained mediation, also known as sequential mediation, is a statistical approach where an independent variable affects a dependent variable through a sequence of mediator variables (Imai et al., 2010). This research is intended to provide theoretical references and practical insights for mitigating negative emotional states in college students. Consequently, the hypotheses of this study include H1: Physical activity can significantly negatively predict negative emotions among college students; H2: Psychological resilience mediates the relationship between physical activity and the negative emotions of college students; H3: Coping styles mediate the relationship between physical activity and college students’ negative emotions; H4: Psychological resilience and coping styles play a chained mediating role between physical activity and negative emotions in college students. The proposed model for our research hypothesis is depicted in Figure 1.

## Methods

### Study population and sampling method

The study population was selected using a stratified random sampling method. Three cities (Hangzhou, Nanjing, and Wenzhou) were randomly selected from the East China region, and then five colleges were randomly selected from these three cities. The survey was conducted from September 2022 to December 2022. According to the sample size calculation method (Preacher and Kelley, 2011), the estimated sample size ranges from 380 to 759. We expanded our analysis to include a larger sample of 1,420 undergraduate students from freshman to senior year.

### Recruitment

The study included participants who met the following criteria: (1) aged between 17 and 25 years old, and (2) enrolled as undergraduate students at the university. The focus on undergraduate students was attributed to their unique developmental stage and specific academic pressures. Additionally, physical education classes, which are mandatory only for undergraduates, are not typically a part of graduate programs in China. This focus also allowed us to maintain sample homogeneity and data consistency, thereby improving the reliability and validity of our results. All participants provided informed consent and participated in the survey voluntarily. The study excluded: (1) graduate students and intern students at the school, and (2) individuals employed by the schools.

### Pilot survey

An initial survey was administered to eight college students across various academic levels to verify the questionnaire’s validity. The primary aim of the pilot survey was to validate the comprehensibility and effectiveness of our questionnaire. We aimed to ensure that the questionnaire’s questions could be widely understood and effectively gather the information required for our research. Based on the respondents’ feedback, suitable modifications were made to the questionnaire.

### Data collection

The investigators, all of whom are public physical education teachers at these universities, received unified training via Tencent meetings. After obtaining approval from the school leadership, they collaborated to complete the questionnaire collection. The questionnaires were collected through face-to-face interviews. Before data entry, errors were identified and corrected, and missing responses were completed, ensuring logical consistency. Questionnaires with clear logical inconsistencies and numerous missing responses were excluded. This approach aimed to enhance the precision of variable measurements within each subgroup. The selected colleges served as the targets for the survey.

### Measurement instruments

#### Physical activity rating scale (PARS-3)

Physical activity was measured using the Physical Activity Rating Scale (PARS-3), developed by Japanese scholar Koshio Hashimoto and modified by Deqing Liang et al. (Liang and Liu, 1994). The scale uses a Likert 5-point scale with three dimensions: exercise intensity, time, and frequency. A 5-point Likert scale is a psychometric response method that allows respondents to express their level of agreement using the following points: (1) Strongly Disagree, (2) Disagree, (3) Neither Agree nor Disagree, (4) Agree, and (5) Strongly Agree. The scoring formula was: physical activity = exercise intensity × (exercise time − 1) × exercise frequency score, with higher scores indicating greater physical activity. Specifically, scores ≤19 are categorized as low physical activity (LPA), scores between 20 and 42 as moderate physical activity (MPA), and scores ≥43 as vigorous physical activity (VPA). The retest reliability of the scale in this study was 0.678.

#### Connor-Davidson resilience scale (CD-RISC)

We adapted the Connor-Davidson Resilience Scale (CD-RISC) developed by Connor and Davidson and revised by Xiaonan Yu et al. (Yu et al., 2011). The modifications were made to create a culturally relevant and psychometrically sound Chinese version of the scale, ensuring its applicability to Chinese-speaking populations. The scale consists of 25 items: 13 for resilience, four for optimism, and eight for self-improvement. A Likert 5-point scale was used for scoring. The internal consistency (alpha) coefficient of the scale in this study was 0.87.

#### Simplified coping style questionnaire (SCSQ)

The Simplified Coping Style Questionnaire (SCSQ) is a 20-item instrument that was developed by Xie (1998). The SCSQ measures two types of coping strategies: positive coping and negative coping. Positive coping strategies are constructive ways individuals deal with stress, such as seeking social support, practicing relaxation strategies, or problem-solving. Negative coping strategies, on the other hand, are behaviors that might temporarily reduce stress but could have long-term detrimental effects, such as avoidance or turning to alcohol or drugs. Using the Likert 4-level scoring method, ‘not adopted’ is scored 0, ‘occasionally adopted’ is scored 1, ‘sometimes adopted’ is scored 2, and ‘often adopted’ is scored 3. This is used to measure the level of positive and negative coping when an individual encounters stress. The scale has been widely used in China and has demonstrated high reliability and validity. In this study, the alpha coefficient of the positive coping subscale was 0.84, and that of the negative coping style subscale was 0.76.

#### Depression anxiety and stress scale (DASS)

The Depression Anxiety and Stress Scale (DASS-21), originally developed by Lovibond et al. in 1995, was used in this study in its Simplified Chinese version as revised by Gong et al. (2010). The adaptations aimed to ensure cultural relevance and psychometric validity for Chinese-speaking populations. The scale contains 21 items, with seven items each for the three subscales of depression, anxiety, and stress. Each item is scored on a 4-point scale ranging from 0 (“does not match”) to 3 (“always matches”). The score for each subscale is obtained by multiplying the sum of its item scores by two, with higher scores indicating a greater likelihood of experiencing the respective emotion.

### Data processing and analysis

The study’s data analysis was conducted using IBM SPSS 22.0 and the PROCESS V3.5 plug-in, chosen for their robust analytical capabilities, compatibility, and the plug-in’s mediation and moderation analysis features. The variables examined in the correlation and regression analyses were carefully selected based on their relevance to the study’s objectives. These variables were subjected to descriptive analysis to provide a summary of their basic features and distributions. ANOVA was conducted to compare the means of these variables across different groups, providing insights into their relationships. Regression analysis was used to understand the predictive relationships between these variables. To validate the mediating effects between variables, a bias-corrected nonparametric bootstrap 95% confidence interval estimation was chosen. This method, involving 5,000 repetitions of sampling, was selected for its advantages in bias correction, skewness adjustment, versatility, and ease of implementation.

Additionally, given that the data in our study is self-reported, there is a potential for common method bias. During the testing process with college students, we emphasized the anonymity and confidentiality of the questionnaire, and clarified that the data is solely for scientific research purposes, in order to control the sources of common method bias as much as possible. Furthermore, we employed Harman’s single-factor test (Baumgartner et al., 2021) to check for common method bias.

## Results

### Data collection and response rates

From September 2022 to December 2022, this survey-based, cross-sectional study collected data from 1,380 college students across five universities in three cities. Specifically, a total of 1,420 questionnaires were distributed to randomly selected students in physical education classes across these colleges. Of these, 1,397 were recovered, and after excluding invalid questionnaires, 1,380 valid responses remained. The recovery rate was 98.3%, and the validity rate was 97.1%. The high recovery rate can be attributed to the strong emphasis on academic participation in these institutions, and no additional incentives were provided. The respondents included 649 freshmen (47%), 469 sophomores (34%), 193 juniors (14%), and 69 seniors (5%). There were 773 male students (56%) and 607 female students (44%).

### Common method bias test

The results of an unrotated exploratory factor analysis using Harman’s one-factor test extracted a total of 11 factors with eigenvalues >1. The maximum variance explained by a single factor was 23.76%, which is less than the critical criterion of 40%. These results indicate that there is no serious common method bias in this study. Common method bias, if present, could potentially inflate the relationships between variables due to shared method variance, leading to erroneous conclusions. However, our test results alleviate these concerns. The fact that no single factor emerged or accounted for the majority of the covariance among the variables suggests that the relationships observed in our study are likely to be attributed to the constructs under investigation rather than to any potential method bias.

### Impact of academic year and major on negative emotions

In this study, we explored the potential influences of both academic year (encompassing freshman, sophomore, junior, and senior stages) and major (including fields such as medicine, foreign languages, law, computer science, education, psychology, materials engineering, chemistry, pharmacy, electronic information, and others) on the prevalence of negative emotions among college students. We employed analysis of variance (ANOVA) with the aim of discerning any statistically significant disparities in the cumulative scores of negative emotions across diverse academic years and majors. Contrary to our expectations, the results revealed that these factors did not exert a significant impact on the reported intensity of negative emotions, as evidenced by *p*-values of 0.405 for academic year and 0.117 for major. This finding implies a uniformity in the experience of negative emotions across various academic progressions and fields of study. Consequently, our analysis lends support to the hypothesis that the intensity of negative emotions experienced by college students is not significantly influenced by their respective academic year or chosen major.

### The relationship between physical activity, psychological resilience, coping styles, and negative emotions

The means (*M*), standard deviations (*SD*) and correlation matrices for physical activity, psychological resilience, coping styles and negative emotions are given in Table 1. The descriptive results of each variable indicate that the average amount of physical activity among college students is at a low level (Mean = 16.911, SD = 17.108). The large standard deviation suggests a wide range of physical activity levels among the students. This could be due to a variety of factors, such as differences in personal habits, academic workload, or access to sports facilities. Psychological resilience, categorized into tenacity, self-improvement, and optimism, along with coping styles, divided into positive and negative coping, and negative emotions, subdivided into depression, anxiety, and stress, form the core variables of our study. When encountering stress, college students tend to use positive coping strategies (Mean = 36.796, SD = 5.640) than negative coping (Mean = 18.944, SD = 4.585).

**Table 1:** Descriptive and correlation statistics of each variable (*n* = 1,380).

		Mean ± SD	1	2	3	4	5	6	7	8	9	10	11
1	Physical activity	16.911 ± 17.108	1										
2	Psychological resilience	10.553 ± 1.665	0.270^**^	1									
3	Tenacity	3.410 ± 0.574	0.270^**^	0.927^**^	1								
4	Self-improvement	3.682 ± 0.598	0.268^**^	0.937^**^	0.857^**^	1							
5	Optimism	3.461 ± 0.641	0.211^**^	0.894^**^	0.712^**^	0.733^**^	1						
6	Coping styles	/											
7	Positive coping	36.796 ± 5.640	0.166^**^	0.602^**^	0.556^**^	0.591^**^	0.515^**^	1					
8	Negative coping	18.944 ± 4.585	−0.020	0.061^*^	0.085^*^	−0.007	0.089^*^	0.199^**^	1				
9	Negative emotions	35.737 ± 12.658	−0.097^**^	−0.222^**^	−0.170^**^	−0.274^**^	−0.170^**^	−0.187^**^	0.399^**^	1			
10	Depression	11.496 ± 4.393	−0.111^**^	−0.253^**^	−0.201^**^	−0.311^**^	−0.187^**^	−0.213^**^	0.389^**^	0.952^**^	1		
11	Anxiety	11.719 ± 4.305	−0.094^**^	−0.186^**^	−0.132^**^	−0.236^**^	−0.144^**^	−0.155^**^	0.353^**^	0.959^**^	0.872^**^	1	
12	Stress	12.522 ± 4.539	−0.075^*^	−0.199^**^	−0.153^**^	−0.239^**^	−0.156^**^	−0.167^**^	0.404^**^	0.957^**^	0.861^**^	0.883^**^	1

^*^Indicates *p* < 0.05; ^**^indicates *p* < 0.001.

Our correlation statistical analysis (Table 1) reveals significant relationships between physical activity, psychological resilience, coping styles, and negative emotions. Specifically, physical activity is positively correlated with both psychological resilience (*r* = 0.270, *p* < 0.001) and positive coping (*r* = 0.166, *p* < 0.001), but negatively correlated with negative emotions (*r* = −0.097, *p* < 0.001).

Moreover, psychological resilience and positive coping share a significant positive correlation (*r* = 0.602, *p* < 0.001). Within the sub-dimensions of psychological resilience, tenacity (*r* = 0.556, *p* < 0.001), self-improvement (*r* = 0.591, *p* < 0.001), and optimism (*r* = 0.515, *p* < 0.001) all exhibit positive correlations with positive coping.

Psychological resilience is significantly negatively correlated with negative emotions (*r* = −0.222, *p* < 0.001), including its sub-dimensions: depression (*r* = −0.253, *p* < 0.001), anxiety (*r* = −0.186, *p* < 0.001), and stress (*r* = −0.199, *p* < 0.001). Similarly, positive coping is negatively correlated with negative emotions (*r* = −0.187, *p* < 0.001) and its sub-dimensions: depression (*r* = −0.213, *p* < 0.001), anxiety (*r* = −0.155, *p* < 0.001), and stress (*r* = −0.167, *p* < 0.001). While, negative coping is positively correlated with negative emotions (*r* = 0. 399, *p* < 0.001) and its sub-dimensions: depression (*r* = 0. 389, *p* < 0.001), anxiety (*r* = 0. 353, *p* < 0.001), and stress (*r* = 0. 404, *p* < 0.001).

These results suggest that individuals can develop a higher level of psychological resilience through physical activity, enabling them to better handle various challenges. It appears that college students prefer to employ positive coping strategies when dealing with stress and negative emotions. This preference may indicate that during physical activity, individuals tend to adopt positive coping mechanisms, such as seeking support and problem-solving, to minimize the experience of negative emotions.

### The relationship between the amount of physical activity and negative emotions among college students of different genders

Previous studies have highlighted significant gender differences in physical activity levels (McCarthy and Warne, 2022). This study further explores gender differences among these variables, as shown in Table 2. We applied an independent samples *t*-test to compare the gender difference. As shown in Table 2, male students are significantly more physically active than female students (*p* < 0.001). The corresponding Cohen’s d value is 0.453, suggesting a near-medium effect size. This aligns with previous findings suggesting that male students are generally more inclined towards active involvement in physical activities (Sisay, 2021; Xu et al., 2021). These findings underscore the potential benefits of promoting physical activity as a strategy to mitigate negative emotions among college students, particularly in males.

**Table 2:** Analysis of gender differences among variables.

	Gender		*t*	*p*	Cohen’s d
	Male (*n* = 773)	Female (*n* = 607)			
Physical activity	20.175	12.755	8.433	< 0.001^**^	0.453
Psychological resilience	10.628	10.457	1.919	0.055	0.123
Tenacity	3.457	3.350	3.523	< 0.001^**^	0.169
Self-improvement	3.705	3.653	1.630	0.103	0.088
Optimism	3.466	3.455	0.314	0.753	0.016
Coping styles					
Positive coping	30.308	31.115	−3.174	0.002^*^	0.173
Negative coping	23.980	23.299	2.193	0.028^*^	0.118
Negative emotions	37.088	34.016	4.606	< 0.001^**^	0.244
Depression	11.903	10.977	3.989	< 0.001^**^	0.211
Anxiety	12.197	11.110	4.789	< 0.001^**^	0.255
Stress	12.988	11.929	4.417	< 0.001^**^	0.235

^*^Indicates *p* < 0.05; ^**^indicates *p* < 0.001. For Cohen’s d: 0.2 is a small effect, 0.5 is a medium effect, and above 0.8 is a large effect.

Regarding coping styles, significant differences were observed between male and female students in both positive (*p* = 0.002, Cohen’s d = 0.173) and negative coping (*p* = 0.028, Cohen’s d = 0.118) (Table 2). Specifically, female students tended to employ more positive strategies to manage stress, aligning with the general characteristics of emotional expression and support-seeking in female students (Chaplin, 2015). These results indicate that although the effect sizes of positive and negative coping strategies are small to tiny, they are still significant in terms of gender differences, suggesting that gender differences in coping strategies may have important implications for stress management and emotional regulation.

The study also found that male students’ overall negative emotion scores were significantly higher than female students (*p* < 0.001) (Table 2). Further analysis revealed significant gender differences in the dimensions of negative emotions, specifically depression (*p* < 0.001, Cohen’s d = 0.211), anxiety (*p* < 0.001, Cohen’s d = 0.255) and stress (*p* < 0.001, Cohen’s d = 0.235). These Cohen’s d values reflect “small” to “near-medium” effect sizes for gender differences in depression, anxiety, and stress. This finding corroborates past studies, suggesting that male students may be less likely to express emotions, leading to an accumulation of negative emotions (MacArthur, 2019; Sagar-Ouriaghli et al., 2020). It further underscores the importance of taking into account the extent and nuances of gender differences in the influence on negative emotions when formulating mental health interventions for college students.

### Relationships between physical activity, psychological resilience, positive coping styles and negative emotions: a mediation effect test

In developing our model, we considered cities and colleges as potential random effect factors, hypothesizing they might influence the dependent variable—total negative emotions score. However, our analysis revealed that the variance components for each random effect were zero. This suggests that the influence of cities and colleges as random factors is either extremely limited or virtually non-existent, indicating a consistent influence across different locations and institutions on the dependent measures.

To further understand the underlying mechanisms affecting negative emotions, we tested the mediating effects of psychological resilience and positive coping styles in the relationship between physical activity and negative emotions. We used Model 6 in SPSS PROCESS V3.3 (Hayes, 2013) to conduct the analyses and showed the results in Table 3.

**Table 3:** Analysis of regression relationship among variables.

	Path	Effect(β)	*SE*	*t*	*p*	LLCI	ULCI
Direct effect	Physical activity→Negative emotions	−0.040	0.029	−1.403	0.161	−0.096	0.016
Indirect effect	Physical activity→Psychological resilience	0.255	0.028	9.126	<0.001^**^	0.200	0.310
	Physical activity→Positive coping	0.027	0.022	1.078	0.281	−0.022	0.075
	Psychological resilience→Positive coping	0.555	0.024	23.087	<0.001^**^	0.508	0.602
	Psychological resilience→Negative emotions	−0.174	0.033	−5.239	<0.001^**^	−0.240	−0.109
	Positive coping→Negative emotions	−0.107	0.033	−1.403	0.161	−0.096	0.016
Total effect	Physical activity→Negative emotions	−0.102	0.028	−3.601	<0.001^**^	−0.158	−0.047

^*^Indicates *p* < 0.05; ^**^indicates *p* < 0.001.

#### Direct effects analysis

Physical activity demonstrated a direct, albeit non-statistically significant, influence on negative affect (effect size (*β*): −0.040, *t* = −1.403, *p* = 0.161). This indicates the potential for physical activity to mitigate negative emotions, possibly through the involvement of other mediating variables.

#### Indirect effects analysis

We employed the bias-corrected nonparametric percentile Bootstrap method to examine the indirect influence of physical activity on negative emotions via psychological resilience. Physical activity was found to have a significant indirect positive effect on negative emotions through psychological resilience (*β* = 0.255, *t* = 9.126, *p* < 0.001). This suggested that psychological resilience can serve as a beneficial psychological resource, aiding individuals in better managing life’s stresses and dilemmas, and consequently reducing the incidence of negative emotions.

However, the indirect effect of physical activity on positive coping was not significant (*β* = 0.027, *t* = 1.078, *p* = 0.281), implying that other mediators might play a more crucial role in the process of physical activity fostering positive coping. Furthermore, psychological resilience demonstrated a significant positive indirect effect on positive coping (*β* = 0.555, *t* = 23.087, *p* < 0.001), underscoring the pivotal role of psychological resilience in promoting positive coping.

Psychological resilience exhibited a negative indirect effect via negative emotions (*β* = −0.174, *t* = −5.239, *p* < 0.001), suggesting that a deficiency in psychological resilience might be linked to increased negative emotions. While, positive coping demonstrated a non-significant negative indirect effect through negative emotions (*β* = −0.107, *t* = −1.403, *p* = 0.161), implying that positive coping strategies might not always succeed in diminishing negative affect.

#### Total effects analysis

The total effects analysis revealed a significant negative correlation (*β* = −0.102, *t* = −3.601, *p* < 0.001) between physical activity and negative emotions (Table 3). This suggests that physical activity could alleviate negative emotions by enhancing psychological resilience and coping styles. This outcome substantiates the validity of the hypothesized model H1.

#### Chain mediating effect analysis

The study examined the mediating effects of physical activity on negative emotions through psychological resilience. Although the direct effect of physical activity on negative emotions was negative, the path analysis results revealed that this negative effect was further amplified when mediated through psychological resilience (effect value: −0.0324, as shown in Table 4). In essence, physical activity was found to reduce the experience of negative emotions by enhancing psychological resilience. This finding is consistent with the theoretical perspective that physical activity can improve an individual’s ability to manage life stress and emotional distress, thereby reducing the intensity of negative emotions (Stults-Kolehmainen and Sinha, 2014; Schultchen et al., 2019). These results provide empirical support for Hypothesis H2 of the model.

**Table 4:** Mediating effect test among variables.

Path	Effect value	Bootstrap SE	Bootstrap 95%CI	
			LLCI	ULCI
Physical activity→Psychological resilience→Negative emotions	−0.0324	0.0084	−0.0503	−0.0173
Physical activity→Positive coping→Negative emotions	−0.0002	0.0014	−0.0033	0.0026
Physical activity→Psychological resilience→Positive coping→Negative emotions	−0.0099	0.0041	−0.0186	−0.0023

The mediating influence of physical activity on negative emotions through positive coping was further examined. However, path analysis revealed that the effect of physical activity on negative emotions, when mediated through positive coping, was not significant. Despite the small mediation effect size (effect value: −0.0002), its 95% confidence interval (−0.0033, 0.0026) encompasses zero. This could suggest the presence of other unconsidered mediating factors in the relationship between these two variables. These findings did not validate the hypothesis H3 of the model. The practical implications of these findings suggest that while positive coping may not significantly mediate the relationship between physical activity and negative emotions, it remains an important factor to consider in mental health interventions.

The study further examined the chain-mediated effect of physical activity on negative emotions through psychological resilience and positive coping. The results indicated a significant chain mediation effect on negative emotions (effect value: −0.0099). This underscores the influence of physical activity on negative emotions under the combined impact of psychological resilience and positive coping, thereby validating the hypothesis H4 of the model. This chain mediation effect provides a more comprehensive understanding of the relationship between physical activity and negative emotions, highlighting the importance of both psychological resilience and positive coping in this relationship.

**Figure 2:**
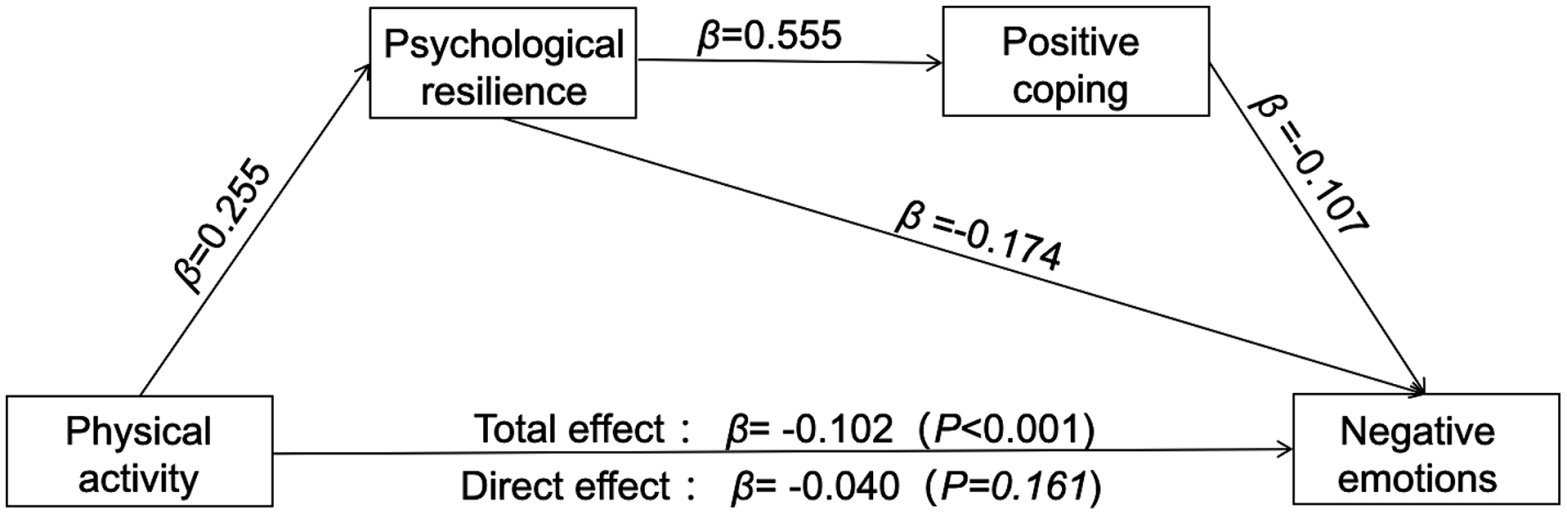
The chain mediating model between physical activity and negative emotions among college students.

The overall chain mediation model between physical activity and negative emotions among college students is depicted in Figure 2. The findings highlight the pivotal role of psychological resilience in mitigating negative emotions, suggesting that interventions for negative emotions can be made through psychological resilience and positive coping.

## Discussion

Our findings suggest that physical activity indirectly serves as a substantial mitigating factor for negative emotions. The average level of physical activity among college students is relatively low, with a tendency towards using positive coping strategies when faced with negative emotions. Notably, our study found a positive correlation between the amount of physical activity and both psychological resilience and positive coping. Conversely, physical activity was negatively correlated with negative emotions, specifically depression, anxiety, and stress. Gender differences were also observed in our study. Male students were significantly more physically active than their female counterparts. However, female students exhibited higher levels of positive coping. In terms of negative emotions such as depression, anxiety, and stress, male students scored significantly higher than female students. Physical activity was found to have a significant overall effect on negative emotions. Furthermore, both psychological resilience and positive coping were identified as chain mediators in the relationship between physical activity and negative emotions.

### The relationship between physical activity and negative emotions

Previous research has primarily employed scale measurements and experimental methods to gather data and quantitatively evaluate the impact of various physical activity programs and the extent of physical activity on the negative emotions of college students (Stults-Kolehmainen and Sinha, 2014; Churchill et al., 2021). These studies have proposed intervention strategies and methods, demonstrating that active physical activity can alleviate negative emotions, enhancing students’ ability to cope with stress and bolstering their psychological resilience. Our study, however, concentrates on the direct and indirect effects of physical activity on the negative emotions of college students. It elucidates the relationship, mechanisms, and effects of physical activity on negative emotions by incorporating elements of psychological resilience and positive coping styles. The findings indicate a significant negative and indirect correlation between physical activity and negative emotions in college students. The total effect of physical activity significantly predicts a decrease in negative emotions, as evidenced in Table 3, thereby confirming Hypothesis H1.

### Mediating effects of psychological resilience

Stressful emotions can disrupt an individual’s internal balance, leading to a reconfiguration of perceptions and a variety of outcomes. Psychological resilience, a beneficial personality trait, serves as a crucial safeguard. It empowers individuals to withstand stress, swiftly adapt to their environment, and mitigate the harmful effects of adverse events (Troy et al., 2023). In the context of college students, psychological resilience plays a pivotal role in preserving their physical and mental equilibrium when they face stressful situations (Gong et al., 2023). Individuals with lower levels of psychological resilience may experience physical discomfort (e.g., shortness of breath, rapid heartbeat, dizziness), mental depression, and cognitive blankness when dealing with stress (Doan et al., 2023).

Psychological resilience can be bolstered through various means, such as self-improvement, setting appropriate life goals, moderating expectations, and maintaining harmonious family relationships. These strategies can assist college students in maintaining a positive psychological state (Wu et al., 2020; Kuang et al., 2024). However, the specific mechanisms through which physical activities strengthen psychological resilience and alleviate negative emotions have not been thoroughly studied.

Psychological resilience, a multidimensional construct with hierarchical characteristics, has been less explored in previous research, which has primarily focused on goals, emotions, and cognition (Chaplin, 2015; Zhang et al., 2022). This study aims to delve into three dimensions of resilience: individual self-improvement, optimism, and resilience, using a comprehensive scale of 25 items. Our findings reveal a robust positive correlation between physical activity and psychological resilience, a correlation that persists even when other control factors are considered. We extend the scope of previous research by demonstrating that physical activity primarily alleviates negative emotions through the comprehensive mediating role of psychological resilience. The divergence between our findings and those of previous studies (Wu et al., 2020; Xu et al., 2021; Gong et al., 2023) underscores the importance of considering the multidimensional nature of psychological resilience in future research, as the differences may be attributed to the varied scales used to measure psychological resilience.

This study affirms Hypothesis H2, indicating that individuals who engage in high levels of physical activity demonstrate increased resistance to negative emotions under stressful circumstances. This finding is consistent with the perspective that psychological resilience is a dynamic process influenced by various factors such as optimism, resilience, and self-improvement (Godara et al., 2022; Ijntema et al., 2023). These traits equip individuals with the capacity to adopt a more constructive problem-solving approach, thereby reducing the negative impact of risk factors and promoting individual adaptation and growth. This underscores the importance of physical activity in fostering psychological resilience and managing stress effectively.

### Mediating effects of positive coping

Coping strategies are generally categorized into positive and negative types. Positive coping acts as a regulatory mechanism for stress, facilitating adaptive responses, whereas negative coping is often characterized by emotional distress and avoidance behaviors (Fteiha and Awwad, 2020). Our findings align with previous research styles (Moeller et al., 2020; Chen et al., 2022), indicating that regular physical activity is significantly associated with the adoption of positive coping. However, it appears to have no significant association with negative coping strategies, as indicated in Table 1. Engaging in physical activity is not merely an exercise but a proactive process that nurtures positive coping mechanisms. Despite this, our current study did not support the hypothesis (H3) that physical activity mitigates negative emotions in college students by significantly mediating through positive coping strategies, as shown in Table 3.

The sustained practice of physical activity has the potential to bolster psychological resilience and improve coping strategies among college students (Xu et al., 2021; Chen et al., 2022). This development promotes an active and engaged approach to challenges, as opposed to a passive or avoidance-based one. It empowers students to utilize their cognitive resources and physical vigor, to actively recalibrate their self-perception, regulate their emotions, and tackle obstacles directly. Thus, it is plausible that physical activity may enhance psychological resilience, thereby transforming their coping strategies.

### Chain-mediating effects of psychological resilience and positive coping

Stressful events inevitably impact individuals, often leading to negative emotions and impaired cognitive functions. However, individuals with higher psychological resilience are more likely to employ positive coping strategies to address these issues. Prior research has indicated that factors such as interpersonal support, peer relationships, and sports friendships at school can foster a willingness to exercise among college students, thereby enhancing their physical activity behavior (Zhang et al., 2022; Zou et al., 2023). Additionally, family parental support, social support, and teacher support can also bolster college students’ motivation and engagement in physical activity. These factors can aid college students in strengthening their commitment to exercise, implementing exercise behaviors, and fostering psychological resilience. This, in turn, enables them to adopt positive coping styles when faced with stress, thereby mitigating negative emotions and other mental health issues.

Our study’s findings suggest that psychological resilience can inspire college students to maintain an optimistic attitude, perseverance, and self-improvement efforts in the face of adversity. They can positively adjust their cognition, confront the problem rather than evade it, and utilize their resources and skills to manage difficulties and alleviate negative emotions. This study further corroborates the fully mediating role of psychological resilience and positive coping styles between physical activity and negative emotions among college students. In other words, physical activity primarily mitigates negative emotions by enhancing psychological resilience and positive coping styles. Thus, hypothesis H4 is validated.

Our study also found significant gender differences in the emotional responses of college students, aligning with previous research (Chaplin, 2015; Graves et al., 2021; McCarthy and Warne, 2022). Female students demonstrated higher levels of positive coping, while male students exhibited more negative emotions, including anxiety, depression, and stress. Notably, male students’ overall negative emotion scores were significantly higher than those of female students. These gender differences have practical implications for designing interventions or support programs for male and female students. For male students, an increased focus can be placed on emotion management and stress coping. This could include counseling services and training programs that enhance their emotional regulation skills. For female students, the emphasis could be on fostering the development of positive coping mechanisms. This might involve promoting positive thinking, self-affirmation, and enhancing self-efficacy.

This study underscores the importance of focusing on the physical activity behavior of college students. It advocates for attention to the university physical education curriculum, calling for curriculum reform and innovation, and the provision of optimal physical education courses. The study further emphasizes the need for qualified physical education teachers who can guide students and help them realize the benefits of physical activity in terms of physical fitness, personality development, and willpower. In addition, the study suggests the implementation of specialized courses tailored to students of different genders. These courses could provide customized exercise programs and emotional management strategies to cater to their unique needs. By understanding and addressing these gender differences, colleges can more effectively support the mental health and well-being of all students. This approach fosters a healthier and more inclusive campus environment, thereby enhancing the overall student experience.

### Limitations and prospects

While this study offers valuable insights into the relationship between physical activity, psychological resilience, positive coping styles, and negative emotions, it’s important to interpret the results with caution due to certain limitations. The self-report method used to measure the intensity, duration, and frequency of participants’ daily physical activity may not accurately represent their actual levels. Future research could benefit from more objective data collection methods, such as using wearable electronic devices. Additionally, this cross-sectional study limits the understanding of potential causal relationships over time. A longitudinal design in future studies could provide more comprehensive insights. Furthermore, potential confounding variables, such as financial means, access to resources, parental support, and upbringing, could influence the observed relationships. Future research should consider these variables for a more holistic understanding of the interplay between physical activity, coping skills, and emotional well-being.

### Suggestions and strategies

#### Reducing pressure and encouraging physical activity

The education department should take appropriate measures to reduce the pressure on college students. Concurrently, the sports department should encourage students to engage in physical activity. Attention should be paid to the quality of physical education classes in colleges and universities, and students should be encouraged to participate in extracurricular physical activities.

#### Developing a campus ‘sports culture’

Developing a ‘sports culture’ on campus is important. However, it’s essential to recognize that physical activity can take many forms outside traditional sports settings. Activities like walking clubs, yoga classes, and other recreational options can appeal to a wider range of individuals. Offering diverse and inclusive options that cater to different interests and preferences can encourage more campus engagement in physical activity.

#### Promoting student health

Several key strategies emerge to promote student health in a university setting. Firstly, promoting active lifestyles through increased physical activity is crucial. This not only fosters healthier habits but also provides a natural outlet for stress and negative emotions. Secondly, developing mental resilience can significantly enhance a student’s ability to cope with the pressures of university life. This can be achieved through targeted training and support, equipping students with effective coping strategies such as positive thinking and problem-solving.

#### Addressing gender differences and fostering community

Recognizing and addressing gender differences is another important aspect. Tailored interventions, such as specific exercise programs and emotional management strategies for female students, can provide more targeted support. Creating a healthy and active environment on campus, with a strong emphasis on sports culture, can further encourage participation in physical activities. Finally, establishing a student mutual support network can foster a sense of community and mutual support. These strategies, when implemented effectively, can significantly improve the overall health and well-being of college students.

## Conclusion

In the context of increasing mental health concerns among college students, our study reveals a significant indirect association between physical activity and the mitigation of negative emotions such as depression, anxiety, and stress. This relationship is mediated by psychological resilience and positive coping styles. These findings contribute to the existing literature, providing a theoretical basis for practical interventions and emphasizing the importance of regular physical activity in improving the overall health and well-being of college students. We recommend incorporating physical activity promotion into existing educational programs and advocate for further research into the relationship between physical activity and mental health.

## Data availability statement

The raw data supporting the conclusions of this article will be made available by the authors, without undue reservation.

## Ethics statement

The studies involving human participants were reviewed and approved by the Ethics Committee of Xiamen Medical College. All participants gave their written informed consent to participate in this study.

## Author contributions

ML: Conceptualization, Data curation, Formal analysis, Investigation, Methodology, Software, Visualization, Writing – original draft, Writing – review & editing. HL: Conceptualization, Investigation, Methodology, Supervision, Validation, Writing – review & editing, Writing – original draft. ZQ: Data curation, Formal analysis, Validation, Writing – review & editing. YT: Software, Writing – review & editing. WY: Resources, Writing – review & editing. RL: Project administration, Resources, Supervision, Validation, Writing – review & editing.
